# Combining Genetic Variants to Improve Risk Prediction for NAFLD and Its Progression to Cirrhosis: A Proof of Concept Study

**DOI:** 10.1155/2018/7564835

**Published:** 2018-03-14

**Authors:** Umberto Vespasiani-Gentilucci, Chiara Dell'Unto, Antonio De Vincentis, Andrea Baiocchini, Marco Delle Monache, Roberto Cecere, Adriano Maria Pellicelli, Valerio Giannelli, Simone Carotti, Giovanni Galati, Paolo Gallo, Francesco Valentini, Franca Del Nonno, Davide Rosati, Sergio Morini, Raffaele Antonelli-Incalzi, Antonio Picardi

**Affiliations:** ^1^Internal Medicine, Geriatrics, and Hepatology Unit, University Campus Bio-Medico, Rome, Italy; ^2^Laboratory of Pathology of The National Institute for Infectious Diseases, Lazzaro Spallanzani, Rome, Italy; ^3^Hepatology Outpatient Clinic, Colleferro Hospital, Rome, Italy; ^4^Liver Disease Unit, San Camillo-Forlanini Hospital, Rome, Italy; ^5^Laboratory of Microscopic and Ultrastructural Anatomy, CIR, University Campus Bio-Medico, Rome, Italy; ^6^University La Sapienza of Rome, Rome, Italy

## Abstract

**Background & Aims:**

Identifying NAFLD patients at risk of progression is crucial to orient medical care and resources. We aimed to verify if the effects determined by different single nucleotide polymorphisms (SNPs) could add up to multiply the risk of NAFLD and NASH-cirrhosis.

**Methods:**

Three study populations, that is, patients diagnosed with NASH-cirrhosis or with noncirrhotic NAFLD and healthy controls, were enrolled. PNPLA3 rs738409, TM6SF2 rs58542926, KLF6 rs3750861, SOD2 rs4880, and LPIN1 rs13412852 were genotyped.

**Results:**

One hundred and seven NASH-cirrhotics, 93 noncirrhotic NAFLD, and 90 controls were enrolled. At least one difference in allele frequency between groups was significant, or nearly significant, for the PNPLA3, TM6SF2, and KLF6 variants (*p* < 0.001, *p* < 0.05, and *p* = 0.06, resp.), and a risk score based on these SNPs was generated. No differences were observed for SOD2 and LPIN1 SNPs. When compared to a score of 0, a score of 1-2 quadrupled, and a score of 3-4 increased 20-fold the risk of noncirrhotic NAFLD; a score of 3-4 quadrupled the risk of NASH-cirrhosis.

**Conclusions:**

The effects determined by disease-associated variants at different* loci* can add up to multiply the risk of NAFLD and NASH-cirrhosis. Combining different disease-associated variants may represent the way for genetics to keep strength in NAFLD diagnostics.

## 1. Introduction

Nonalcoholic fatty liver disease (NAFLD) is becoming an epidemic in Western populations, with prevalence ranging from approximately 20% to 30% depending on the country and the different reports [1, 2]. Fortunately, against these alarming numbers, only a minority of NAFLD patients evolve to cirrhosis and eventually to hepatocellular carcinoma (HCC), while the majority of them present a relatively benign disease, usually integrated in a dysmetabolic profile [3]. Identifying those patients at risk of progression would be crucial to orient medical care and economic resources, maximizing the benefits of specific hepatologic follow-up and interventions.

A growing body of evidence is demonstrating that genetic susceptibility plays an important role in NAFLD development and evolution [4]. Indeed, the role of genetic variation in NAFLD, specifically single nucleotide polymorphisms (SNPs), has been the focus of extensive research in the last decade. The nonsynonymous rs738409 C/G variant in patatin-like phospholipase domain containing protein 3 (PNPLA3) is considered the major genetic component of NAFLD [5]. More recently, the nonsynonymous rs58542926 variant located in the transmembrane 6 superfamily member 2 (TM6SF2) gene has emerged as the second genetic determinant of NAFLD in terms of importance [6, 7]. Many other SNPs of different genes involved in lipid metabolism, such as lipin 1 (LPIN1), oxidative stress, such as superoxide dismutase 2 (SOD2), and fibrogenesis, such as Kruppel-like factor 6 (KLF6), have been associated with the severity of liver damage in different studies on NAFLD patients [8–12]. Altogether, it seems reasonable to assume that no single gene variant can suffice to accurately stratify the risk, while the predictive power would be enhanced by analyzing different SNPs or combining their effect in a genetic risk score. A proof of concept that genetic scores may be useful in NAFLD patients has been already given by Nobili et al. in the pediatric population, where a 4-polymorphism risk score was demonstrated to predict nonalcoholic steatohepatitis (NASH) [13]. More recently, the only similar experience in the adult population was obtained in a Mexican series of morbid obese patients undergoing bariatric surgery, where a genetic risk score including 4 SNPs was associated with hepatic triglyceride and cholesterol content [14]. However, to our knowledge, no attempt has been made to verify the capability of a genetic risk score to stratify the risk of NAFLD and of its evolution to cirrhosis.

Based on this background, in the present study, we aimed to verify if combining different SNPs in a genetic score could increase the predictive power for more severe forms of NAFLD in adult patients. To address this, we performed a case-control study in a well-characterized NAFLD population and tested the same SNPs which had been previously assembled to provide the genetic score in the pediatric population [13], plus TM6SF2, which has more recently emerged as a genetic key determinant of NAFLD [6, 7]. A control group representing the general healthy population was also included.

## 2. Patients and Methods

### 2.1. Patients

Patients followed at the Hepatology Unit of the University Hospital Campus Bio-Medico of Rome, at the Hepatology outpatient clinic of the Colleferro Hospital (Rome, Italy), and at the Liver Disease Unit of the San Camillo-Forlanini Hospital of Rome and those assessed at the Laboratory of Pathology of the National Institute for Infectious Diseases Lazzaro Spallanzani of Rome and recorded in the respective clinical databases with the diagnosis of NASH cirrhosis were extracted. When reviewing the clinical records for the purpose of the present study, the etiological diagnosis of cirrhosis as post-NASH was considered definite and patients eligible for the study only after having verified that all the other causes of liver disease had been excluded and that at least one of the two criteria was satisfied, (1) histological diagnosis of NASH cirrhosis; (2) clinical diagnosis of cirrhosis in patients with a previous documented history of steatosis (by abdominal ultrasonography, computed tomography scan, or magnetic resonance imaging) and at least one of the following diagnoses precede that of cirrhosis: type-2 diabetes mellitus (T2DM), obesity with a body mass index (BMI) > 30 kg/m^2^, and metabolic syndrome. Secondly, the group of patients who had received the diagnosis of noncirrhotic NAFLD (simple steatosis or NASH) by liver biopsy performed at the same Centers was extracted from the clinical databases. Also in this case, it was confirmed that all the other causes of liver disease had been excluded before or after liver biopsy. More specifically, it was verified that none of the patients with NASH cirrhosis and noncirrhotic NAFLD had history of alcoholic intake >20 gr/day if woman and >30 gr/day if man or of use of drugs known to induce liver steatosis. Moreover, it was verified that the following results had been obtained from all the patients: negative anti-hepatitis C virus (anti-HCV) antibodies and hepatitis B surface antigen (HBsAg); anti-nuclear antibodies (ANA) ≤1 : 80 and negative anti-mitochondrial (AMA), anti-smooth muscle (ASMA), and anti-liver and kidney microsomal (anti-LKM) antibodies; normal transferrin saturation and serum levels of ceruloplasmin and alpha-1 antitrypsin. Finally, a third group to represent the general population was recruited among consecutive subjects undergoing general medicine company checkups at the Campus Bio-Medico Hospital. Upper abdomen ultrasonography is among the examination included in every checkup, and ultrasonographic steatosis was considered the only exclusion criterion.

Patients with NASH cirrhosis and noncirrhotic NAFLD individuated according to the aforementioned criteria were contacted and invited to participate to the study, as well as all consecutive subjects attending company checkups. All subjects enrolled in the present study agreed to participate and signed an informed consent. The protocol of the study was approved by the Ethics Committee of the University Campus Bio-Medico of Rome.

### 2.2. Methods

Epidemiological, anthropometric, and clinical data at the time of cirrhosis diagnosis of liver biopsy or of the medical checkup were collected for patients with NASH cirrhosis and noncirrhotic NAFLD and for subjects of the control group, respectively. All subjects enrolled in the study underwent blood drawing and venous blood was stored at −80° until examination.

#### 2.2.1. PNPLA3 rs738409, TM6SF2 rs58542926, KLF6 rs3750861, SOD2 rs4880, and LPIN1 rs13412852 Genotyping

Genomic DNA was extracted from whole blood using the kit DNA-Sorb-B (Sacace Biotechnologies, Como, Italy), as instructed by the manufacturer. The analysis of the five polymorphisms was done by real-time polymerase chain reaction (rt-PCR) using a commercial kit (FLT PLUS Fatty Liver Test; Orga Bio Human, Rome, Italy), as instructed by the manufacturer. For each SNP, the presence of two TaqMan® allows allelic discrimination (ancestral allele, variant allele, or both).

#### 2.2.2. Hepatic Ultrasonography and Liver Biopsy

Abdominal ultrasound was performed with an Acuson S3000 ultrasound system (Siemens®, Munich, Germany), using both a 1.5–6 MHz curved array probe and a 5.5–18 MHz high frequency linear array probe, the latter for the evaluation of the left liver surface. All patients were studied with subcostal, right intercostal and transaxial epigastric scans, and ultrasonography was operated by a single experienced liver sonographer blinded to the patients' clinical data. Liver was defined bright when abnormally intense, high-level echoes arose from hepatic parenchyma and/or when a liver-kidney difference in echo amplitude was detected [15]. The ultrasonographic diagnosis of liver cirrhosis was suggested, to be clinically confirmed by combination with biochemical ± upper endoscopic ± transient elastographic findings, in the presence of nodular liver surface in the left lobe by the high frequency probe and at least one of the following signs of portal hypertension: splenomegaly, dilatation of the main portal, portal-systemic collaterals, and ascites [16, 17].

Liver biopsy was performed under ultrasonographic guidance, and specimens (at least 1.5 cm in length) were fixed in formalin for evaluation. Tissue sections were stained with hematoxylin and eosin and with Sirius red. Liver biopsies were reviewed by two expert liver pathologists (A. B. and S. C.), who were unaware of clinical and genetic data. Histological cases of NASH cirrhosis were reevaluated in order to confirm diagnosis, while cases of noncirrhotic NAFLD were diagnosed as simple steatosis or NASH [18] and staged according to Kleiner et al. [19].

### 2.3. Statistical Analysis

Results are reported as mean ± standard deviation (SD), number (%), and OR with 95% confidence intervals (CI), as appropriate. Differences between groups of patients with NASH cirrhosis and noncirrhotic NAFLD and healthy controls were tested using Kruskal-Wallis test for continuous variables and *χ*^2^ test for categorical variables. A genetic score was designed taking into account the PNPLA3, TM6SF2, and KLF6 polymorphisms, and its association with noncirrhotic NAFLD and NASH cirrhosis was estimated by means of logistic regression models. Models were finally adjusted for age, sex, BMI, and diabetes mellitus. A *p* < 0.05 was considered statistically significant. All analyses were carried out with R 3.3.3 for Mac (R Foundation).

## 3. Results

### 3.1. Study Population

All subjects included in the present study were of Caucasian race and of Italian nationality. One hundred and seventy-five patients with the diagnosis of NASH cirrhosis were extracted from the four clinical databases. Among these patients, 35 had died, 17 refused to participate to the study, and other 16 were discarded, 12 because they did not meet the aforementioned criteria to be included as NASH cirrhosis cases and 4 because of an excessive alcohol intake. Finally, 107 patients with NASH cirrhosis were enrolled: 39 had received histological diagnosis of NASH cirrhosis; 68 had received the clinical diagnosis of cirrhosis (clinical, biochemical, and ultrasonographic ± upper endoscopic ± transient elastographic findings) and, according to inclusion criteria, had a previous documented history of steatosis (mainly ultrasonographic) together with at least one among T2DM (63 patients), obesity (53 patients), and metabolic syndrome (60 patients) preceding the diagnosis of cirrhosis. One hundred and nineteen patients who had received the histological diagnosis of noncirrhotic NAFLD were extracted from the four clinical databases. Five patients had died, 13 of them refused to participate in the study, and 8 were discarded, 5 because of an excessive alcohol intake and three because not all the alternative etiologies had been excluded. Finally, 93 patients with noncirrhotic NAFLD were enrolled in the study. According to Brunt et al. [18], 15 cases had the criteria for simple steatosis and 78 for NASH, and fibrosis was classified as F0, F1, F2, and F3 in 9, 42, 22, and 20 cases, respectively. One hundred and ten consecutive subjects undergoing general medicine company checkups at the Campus Bio-Medico Hospital were evaluated for enrollment as controls. After having excluded 20 subjects due to the finding of ultrasonographic bright liver, 90 subjects were finally recruited since there were no refusals to participate in the study in this group. These subjects were selected to represent the genetic profile in the general population and, therefore, there were no other exclusion criteria.

### 3.2. Epidemiologic and Clinical Data and Frequency of the Five Different SNPs Analyzed

As reported in Table 1, while gender distribution was not different between the two groups, patients with NASH cirrhosis were significantly older than those with noncirrhotic NAFLD. This finding was expected and is consistent with the natural history of NAFLD and of every liver disorder, since fibrosis evolves with disease duration. Also mean BMI and the prevalence of T2DM were significantly higher in patients with NASH cirrhosis with respect to those with noncirrhotic NAFLD. Healthy control subjects presented a gender distribution comparable with that in the other two groups, but they were significantly younger and, as expected considering the dysmetabolic background of the NAFLD population, they had a lower BMI and a lower prevalence of T2DM.

The frequency of the PNPLA3 rs738409 minor (G) allele was significantly higher in patients with NASH cirrhosis than in the other two study populations, as well as in noncirrhotic NAFLD patients with respect to healthy controls. Concerning the TM6SF2 rs58542926 polymorphism, an increase of the T allele frequency was observed in patients with NASH cirrhosis with respect to healthy subjects, while the comparisons between the other groups did not yield statistically significant differences. Similarly to the latter, the increase in the frequency of the T allele at the KLF6 rs3750861* locus* tended to significance when comparing NASH cirrhotics to healthy subjects (*p* = 0.06), while there were no significant differences between the other groups. Finally, the frequencies of the T allele at the SOD2 rs4880* locus* and that of the T allele at the LPIN1 rs13412852* locus* did not show any significant difference when comparing the three groups of subjects analyzed in the study.

Table 3 reports epidemiological, anthropometric, and genetic data concerning the noncirrhotic NAFLD group subdivided into patients with absent/mild (F0-1) and advanced (F2-3) fibrosis. Patients with advanced fibrosis were significantly older, with a higher mean BMI and a higher prevalence of T2DM, with respect to patients with absent/mild fibrosis. Conversely, there were no significant differences in allele frequencies between the two subgroups.

### 3.3. Genotype Distribution at the PNPLA3 rs738409, TM6SF2 rs58542926, KLF6 rs3750861, LPIN1 rs13412852, and SOD2 rs4880 Loci and Derivation of a Genetic Risk Score

Genotype distribution for the polymorphisms of interest at the five different* loci* are reported in Table 2. Concerning PNPLA3, there was a highly significant shift towards the reduction of CC homozygous and the increase of homozygous for the minor G allele (GG) when passing from healthy controls, through noncirrhotic NAFLD patients, to NASH cirrhotics. To note, the different genotype distribution at the PNPLA3* locus* was significant when comparing all groups except for NASH cirrhotics with noncirrhotic NAFLD patients (*p* = 0.06). Genotype distribution for the polymorphisms studied at the TM6SF2 and KLF6* loci* showed a nonsignificant trend towards the reduction of wild-type CC homozygous and the increase of heterozygous carriers of the polymorphic T allele when moving from healthy controls, through noncirrhotic NAFLD, to NASH cirrhotics. In the case of both TM6SF2 and KLF6, when the CT and TT genotypes were grouped, this trend was even stronger and the difference in genotype distribution between NASH cirrhotics and healthy controls became significant for TM6SF2 but still not significant for KLF6 (*p* = 0.09). Conversely, there was not virtually any difference between the three groups in genotype distribution for the SOD2 rs4880 and the LPIN1 rs13412852 polymorphisms. Finally, when genotype distributions of noncirrhotic NAFLD patients were compared between those with absent/mild and those with advanced fibrosis, no significant differences were observed (Table 3).

The PNPLA3 rs738409, TM6SF2 rs58542926, and KLF6 rs3750861 polymorphisms were then considered for the development of the genetic risk score. Consistent with previous literature [7, 11] and according to the Akaike information criterion (data not shown) [20], the models with the best fit for the association with the risk of NASH cirrhosis with respect to noncirrhotic NAFLD were additive for the PNPLA3 polymorphism (CC = 0; CG = 1; GG = 2) and dominant for the TM6SF2 and KLF6 polymorphisms (CC = 0; CT and TT = 1). The relative weight to give to each polymorphism in the computation of the genetic score was determined considering the coefficients of a logistic regression analysis for the candidate genes (modelled as mentioned above) and the presence of NASH cirrhosis (PNPLA3: 0.43; TM6SF2: 0.39; KLF6: 0.46), standardized for their mean and rounded at the unit (PNPLA3: 0.43/0.43 = 1; TM6SF2: 0.39/0.43 = 0.91; KLF6: 0.46/0.43 = 1.06). Accordingly, the genetic risk score was then computed as the sum of one point for each single polymorphism, ranging from 0 to 4.

**Figure 1:**
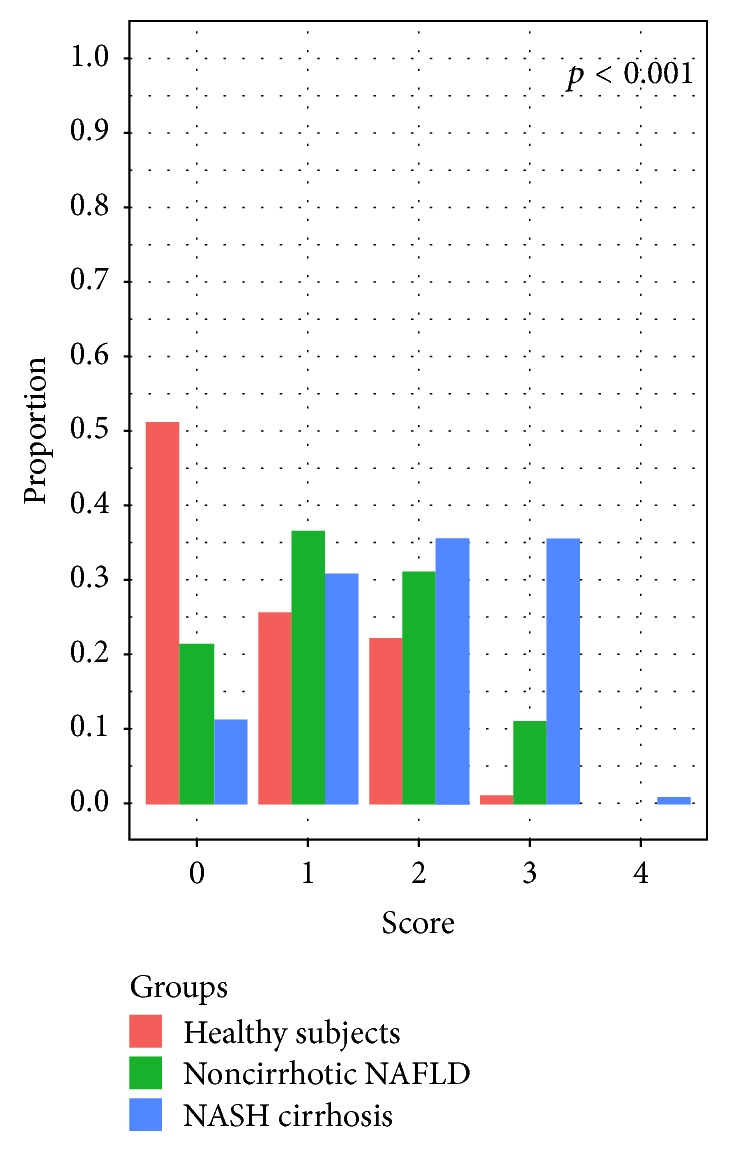
Prevalence of different results in the genetic risk score according to the three study populations.

The genetic risk score was significantly higher in noncirrhotic NAFLD patients with respect to healthy subjects [1.3 (0.9) versus 0.8 (0.9), *p* < 0.001] and in NASH cirrhotics [1.7 (1.0)] when compared to noncirrhotic NAFLD (*p* = 0.005) and to healthy subjects (*p* < 0.001).  Figure 1 shows the percentages of healthy subjects, noncirrhotic NAFLD patients, and NASH cirrhotics presenting the different results of the genetic risk score. Nearly 50% of healthy subjects showed a risk score of 0, while their prevalence decreased progressively with the increase of the score. On the other hand, only about 10% of NASH cirrhotics exhibited a score of 0, the majority of them showing a score of 1 or 2, and about 20% a score ≥ 3.

Table 4 reports univariate and multivariate logistic regression analyses in which the genetic risk score was tested for its association with the risk of noncirrhotic NAFLD and of NASH cirrhosis. When expressed linearly, with healthy subjects as reference group, the genetic risk score was significantly associated with a doubling of the risk of noncirrhotic NAFLD and a tripling of the risk of NASH cirrhosis, both in unadjusted models and in models adjusted for age, sex, BMI, and T2DM. Moreover, with noncirrhotic NAFLD patients as reference group, each point of the score was also associated with an about 50% increased risk of NASH cirrhosis. The results were even more interestingly when the score was expressed categorically. Indeed, when compared to a score of 0, with respect to healthy subjects, a genetic risk score of 1-2 approximately quadrupled and a score of 3-4 increased approximately 20-fold the risk of noncirrhotic NAFLD. Consistently, when compared to a score of 0, with respect to noncirrhotic NAFLD patients, a genetic risk score of 3-4 almost quadrupled the risk of NASH cirrhosis.

## 4. Discussion

This is the first study aimed to verify if combining the presence of different polymorphisms of risk could increase the predictive power for NAFLD and, mainly, for its evolution to cirrhosis. The present results are proof of concept that the effects determined by disease-associated variants at different* loci* can add up to multiply the risk of NAFLD and of NASH cirrhosis.

A growing body of evidence indicates that NAFLD develops as a result of a complex process in which genetic susceptibility plays an important role [21]. According to the available data, the heritability estimates range from 20% to 70%, depending on the study design, ethnicity, and the methodology applied for diagnostic purposes [4]. In the last decade, extensive research deriving from candidate gene studies and, mostly, from genome-wide association studies and exome-wide association studies, has identified several* loci* associated with disease susceptibility and progression. The firstly recognized and still more significant is certainly the rs738409 SNPs in the PNPLA3 gene, encoding for the isoleucine to methionine substitution at position 148 (I148M). Indeed, the I148M variant has been associated with the degree of hepatic injury and all the histopathological aspects of NAFLD, including the presence of NASH, fibrosis, and development of HCC [5, 22]. More recently, we have reported that the rs738409 PNPLA3 SNP is associated not only generically with NAFLD fibrosis but also specifically with the evolution to NASH cirrhosis [23]. To date, the rs58542926 SNP in the TM6SF2* locus* should be considered in the second place of importance. The TM6SF2 variant has been associated with steatosis and fibrosis in patients with NAFLD [24, 25]. Actually, the analysis of patients with chronic HCV infection demonstrated that the rs58542926 SNP enhances liver fibrogenesis also in this setting [26], in which the virus and the host can contribute synergistically to the progression of liver disease and atherosclerosis [27].

American and European NAFLD guidelines recognize the disease modifying role of the PNPLA3 variant and of both the PNPLA3 and the TM6SF2 SNPs, respectively, but testing for these genetic variants in routine clinical practice is still not recommended [2, 28], likely because no single variant has demonstrated sufficient discriminative properties.

A pioneering study aimed to combine the effect of different SNPs in a genetic risk score able to predict NASH has been carried out by Nobili et al. in the pediatric population [13]. In adults, a genetic risk score based on 4 different SNPs has been associated with hepatic lipid content in Mexican morbid obese patients [14]. More recently, a significant increase of serum AST activity and trends for increased ALT and GGT activities have been reported with the increment of risk alleles at 3 loci (rs738409 in PNPLA3; rs58542926 in TM6SF2; rs641738 in membrane bound O-acyltransferase containing 7, MBOAT7) in a German population of adult NAFLD patients [29].

Based on this background, we designed this case-control study in a well-characterized NAFLD population from 4 different hepatological tertiary centers from Rome and its province, by recruiting three groups: NASH cirrhotics, noncirrhotic NAFLD, and controls. This subdivision may have the limit to render the noncirrhotic NAFLD population heterogeneous, since it was constituted by patients with both mild (F0-1) and advanced fibrosis (F2-3). Moreover, since NAFLD patients with milder fibrosis are younger than those with more advanced disease, the possibility that they can progress to advanced fibrosis and even cirrhosis in the following years cannot be ruled out. Actually, all the previously relevant studies on this same topic are affected by this same possible bias [v.b.], which can limited only by a fully adjusted statistics, as that applied in our case. Moreover, whatever cut-off is applied for fibrosis in a cross-sectional study, F0 versus F1-4 [30], F0-1 versus F2-4 [31], F0-2 versus F3-4 [32], or F0-3 versus F4, it should be considered completely arbitrary, and it depends on study population composition and on the objectives of the study itself. Here, by enrolling a large cohort of NASH cirrhotics, we specifically aimed to evaluate the risk of progression to cirrhosis, since cirrhotic evolution, together with the incidence of HCC, should be considered the most clinically relevant outcome in the context of liver disease.

In order to avoid the risk of missing cases of early cirrhosis, noncirrhotic NAFLD patients were included rigorously based on the result of a liver biopsy. Conversely, in agreement with previous studies on NASH cirrhosis and NAFLD-related HCC [33], liver histology was not considered an essential requisite for a NASH cirrhotic patient to be included in the study. The validity of the criteria applied for the diagnosis of NASH cirrhosis is reflected in the clinical characteristics of our population of NASH cirrhotics, who present the typical dysmetabolic profile of most severe forms of NAFLD. A group of patients attending the hospital without any disease-oriented reason, that is, those undergoing general medicine checkups, was finally included. Concerning the SNPs to be tested, we decided to include the 4 which had been included in the original pediatric genetic risk score [13], plus the rs58542926 TM6SF2 variant, which has been more recently acquired as a strong disease modifier in the context of NAFLD [6, 7]. Further than the PNPLA3 and the TM6SF2 SNPs, we therefore tested the rs3750861 KLF6, the rs13412852 LPIN1, and the rs4880 SOD2 SNPs, which have demonstrated associations with fibrosis, steatosis and fibrosis, and again fibrosis, respectively, in previous studies on NAFLD patients [8–12].

Epidemiological and anthropometric data confirmed that age, BMI, and T2DM are strong risk factors for NAFLD and for its fibrotic evolution. Concerning genetics, as expectable, the most significant intergroup differences were observed for the PNPLA3 variant. A clear trend towards an increased prevalence of the minor T allele when moving from healthy subjects, through noncirrhotic NAFLD patients, to NASH cirrhotics, was observed also for the TM6SF2 SNP, for which the difference between NASH cirrhotics and healthy controls was actually significant, and for the KLF6 variant, for which the difference between these same two populations was only at the limit of significance. Conversely, concerning the other two SNPs, the frequency of the minor allele in the three different study populations was almost identical. No significant differences in genetics were observed between noncirrhotic NAFLD patients with absent/mild and those with advanced fibrosis, but the limited number in this subanalysis should be considered. Only the PNPLA3, TM6SF2, and KLF6 SNPs were then selected to constitute the genetic risk score, participating according to the model which best fitted with their association to the risk of NASH cirrhosis. The genetic risk score was significantly associated with the risk of both noncirrhotic NAFLD with healthy subjects as reference group and with the risk of NASH cirrhosis with noncirrhotic NAFLD as reference. The more impressive results were obtained, to our opinion, when the score was expressed categorically; notably, for example, with respect to a score of 0, after adjusting for age, sex, diabetes, and BMI, a score > 2 was associated with about 20-fold increased risk of NAFLD development in healthy subjects and almost 4-fold increased risk of progression to cirrhosis in NAFLD patients.

The strengths of this study are the following: it is carried-out in a well-characterized NAFLD population from hepatological tertiary centers; it includes a control group to represent genetics in the general population; it is the first evident proof of concept that the more the information from genetics the higher possibility to predict the risk of NAFLD and its progression to cirrhosis. This study has also some limitations: it is a case-control study with a transversal design, carrying the possible biases which have been addressed previously; the inclusion of some variants with respect to others which are known to be associated with NAFLD, such as the rs641738 SNP in MBOAT7 [34], can be considered arbitrary. However, it is clear that we did not aim to launch a specific set of variants nor the way to combine them in a genetic risk score; rather, our aim was to give proof of concept that such an approach has a strong logic in the context of NAFLD.

In conclusion, the present study suggests that combining different disease-associated variants may probably represent the way for genetics to keep strength and consent in NAFLD diagnostics. Further studies on larger series are eagerly awaited in order to individuate the best set of variants to be included and the most powerful model and also to verify if combining the best genetic model with clinical predictors would lead to further and meaningful advantages.

## Figures and Tables

**Table 1:** Epidemiologic and clinical data and frequency of the PNPLA3 rs738409 G, TM6SF2 rs58542926 T, KLF6 rs3750861 T, SOD2 rs4880 T, and LPIN1 rs13412852 T alleles in the study population, divided according to subgroups.

	All	Healthy Subjects	Noncirrhotic NAFLD	NASH cirrhosis
*N*	290	90	93	107
Age (years)	54.4 (15.1)	43.1 (11.9)	51.6 (13.2)^++^	66.2 (9.8)^++*∗∗*^
Sex (male)	53.5%	46.6%	60.2%	53.3%
BMI (Kg/m^2^)	28.8 (5.3)	25.2 (4.6)	29.5 (4.1)^++^	31.2 (5.2)^++*∗*^
T2DM	41.6%	7%	45.2%^++^	66.4%^++*∗*^
PNPLA3 G allele frequency	0.46	0.24	0.48^++^	0.61^++*∗*^
TM6SF2 T allele frequency	0.11	0.06	0.11	0.14^+^
KLF6 T allele frequency	0.08	0.06	0.07	0.12
SOD2 T allele frequency	0.5	0.48	0.53	0.49
LPIN1 T allele frequency	0.32	0.35	0.32	0.31

Data are shown as mean (standard deviation) or percentages. ^+^*p* < 0.05 and ^++^*p* < 0.001 versus healthy subjects; ^*∗*^*p* < 0.01 and ^*∗∗*^*p* < 0.001 versus noncirrhotic NAFLD; NAFLD: nonalcoholic fatty liver disease; NASH: nonalcoholic steatohepatitis; BMI: body mass index; T2DM: type 2 diabetes mellitus; PNPLA3: patatin-like phospholipase domain containing protein 3; TM6SF2: transmembrane 6 superfamily member 2; KLF6: Kruppel-like factor 6; SOD2: superoxide dismutase 2; LPIN1: lipin 1.

**Table 2:** Comparison of PNPLA3 rs738409, TM6SF2 rs58542926, KLF6 rs3750861, SOD2 rs4880, and LPIN1 rs13412852 polymorphisms prevalence, according to study subgroups.

	Healthy Subjects	Noncirrhotic NAFLD	NASH cirrhosis	*p*
PNPLA3				
CC	53 (58.9%)	30 (32.3%)^++^	20 (18.7%)^++^	<0.001
CG	30 (33.3%)	36 (38.7%)^++^	44 (41.1%)^++^	
GG	7 (7.8%)	27 (29%)^++^	43 (40.2%)^++^	
TM6SF2				
CC	79 (87.8%)	74 (79.6%)	79 (73.8%)	0.105
CT	11 (12.2%)	17 (18.3%)	27 (25.2%)	
TT	0 (0%)	2 (2.2%)	1 (0.9%)	
TM6SF2 dichotomized				
CC	79 (87.8%)	74 (79.6%)	79 (73.8%)^+^	0.06
CT/TT	11 (12.2%)	19 (20.4%)	28 (26.2%)^+^	
KLF6				
CC	79 (87.8%)	80 (86%)	83 (77.6%)	0.224
CT	11 (12.2%)	13 (14%)	23 (21.5%)	
TT	0 (0%)	0 (0%)	1 (0.9%)	
KLF6 dichotomized				
CC	79 (87.8%)	80 (86%)	83 (77.6%)	0.113
CT/TT	11 (12.2%)	13 (14%)	24 (22.4%)	
SOD2				
CC	26 (28.9%)	20 (21.5%)	30 (28%)	0.795
CT	42 (46.7%)	48 (51.6%)	49 (45.8%)	
TT	22 (24.4%)	25 (26.9%)	28 (26.2%)	
LPIN1				
CC	38 (42.2%)	45 (48.4%)	49 (45.8%)	0.672
CT	41 (45.6%)	37 (39.8%)	50 (46.7%)	
TT	11 (12.2%)	11 (11.8%)	8 (7.5%)	

Data are shown as absolute number and column percentages. *p* for overall chi-square test. ^+^*p* < 0.05 and ^++^*p* < 0.001 versus healthy subjects; PNPLA3: patatin-like phospholipase domain containing protein 3; TM6SF2: transmembrane 6 superfamily member 2; KLF6: Kruppel-like factor 6; SOD2: superoxide dismutase 2; LPIN1: lipin 1.

**Table 3:** Epidemiological, anthropometric, and genetic data of the noncirrhotic NAFLD group divided into two subgroups according to the degree of fibrosis: absent/mild (F0-F1) and advanced (F2-3).

	F0-1	F2-3	*p*
*N*	51	42	
Age (years)	46.7 (13.1)	57.5 (11)	<0.001
Sex (male)	64.7%	54.8%	0.332
BMI (Kg/m^2^)	28.5 (3.8)	30.8 (4.2)	0.012
T2DM	31.4%	61.9%	0.006
PNPLA3			
G allele frequency	0.44	0.54	0.203
CC	19 (37.3%)	11 (26.2%)	0.491
CG	19 (37.3%)	17 (40.5%)	
GG	13 (25.5%)	14 (33.3%)	
TM6SF2			
T allele frequency	0.09	0.14	0.375
CC	42 (82.4%)	32 (76.2%)	0.278
CT	9 (17.6%)	8 (19%)	
TT	0 (0%)	2 (4.8%)	
KLF6			
T allele frequency	0.06	0.08	0.781
CC	45 (88.2%)	35 (83.3%)	0.705
CT	6 (11.8%)	7 (16.7%)	
TT	0 (0%)	0 (0%)	
SOD2			
T allele frequency	0.56	0.49	0.395
CC	9 (17.6%)	11 (26.2%)	0.58
CT	27 (52.9%)	21 (50%)	
TT	15 (29.4%)	10 (23.8%)	
LPIN1			
T allele frequency	0.31	0.32	1
CC	26 (51%)	19 (45.2%)	0.585
CT	18 (35.3%)	19 (45.2%)	
TT	7 (13.7%)	4 (9.5%)	
SCORE (linear)	1.2 (0.9)	1.5 (1)	0.139

Data are shown as mean (standard deviation), percentages, or absolute number and column percentages. NAFLD: nonalcoholic fatty liver disease; NASH: nonalcoholic steatohepatitis; BMI: body mass index; T2DM: type 2 diabetes mellitus; PNPLA3: patatin-like phospholipase domain containing protein 3; TM6SF2: transmembrane 6 superfamily member 2; KLF6: Kruppel-like factor 6; SOD2: superoxide dismutase 2; LPIN1: lipin 1.

**Table 4:** Uni- and multivariable regression models for the association of the genetic score (modelled as numeric or as factor variable) and presence of NASH cirrhosis or noncirrhotic NAFLD.

	NASH cirrhosis versus noncirrhotic NAFLD		Noncirrhotic NAFLD versus healthy controls		NASH cirrhosis versus healthy subjects	
	OR	aOR	OR	aOR	OR	aOR
Score	1.54 (1.14–2.1)	1.51 (1.04–2.22)	2.05 (1.46–2.93)	2.22 (1.40–3.65)	3.01 (2.14–4.38)	3.62 (1.63–9.69)
Score = 0	ref	ref	ref	ref	ref	ref
Score = 1	1.62 (0.69–3.9)	2.07 (0.69–6.45)	3.40 (1.63–7.28)	4.59 (1.74–12.91)	5.50 (2.46–13.00)	15.40 (2.32–149)
Score = 2	2.18 (0.93–5.29)	2.5 (0.84–7.79)	3.33 (1.55–7.36)	4.38 (1.58–13.05)	7.28 (3.24–17.35)	18.54 (2.78–176)
Score = 3	3.83 (1.4–11.14)	3.66 (1.04–13.75)	23.00 (4.01–436.62)	21.98 (2.73–496.88)	88.17 (16.11–1658)	125.37 (4.22–19336)

OR: odds ratio; aOR: adjusted odds ratio. aOR are adjusted for age, sex, BMI, and diabetes mellitus.
